# Defects in the synthetic pathway prevent DIF-1 mediated stalk lineage specification cascade in the non-differentiating social amoeba, *Acytostelium subglobosum*

**DOI:** 10.1242/bio.20148359

**Published:** 2014-05-29

**Authors:** Kurato Mohri, Takashi Hata, Haruhisa Kikuchi, Yoshiteru Oshima, Hideko Urushihara

**Affiliations:** 1 Faculty of Life and Environmental Sciences, University of Tsukuba, 1-1-1 Tennodai, Tsukuba, Ibaraki 305-8572, Japan; 2 Graduate School of Pharmaceutical Sciences, Tohoku University, Aoba-yama, Aoba-ku, Sendai 980-8578, Japan

**Keywords:** Cell differentiation, Cellular slime mold, Polyketide signaling, Gene expression, Functional complementation

## Abstract

Separation of somatic cells from germ-line cells is a crucial event for multicellular organisms, but how this step was achieved during evolution remains elusive. In *Dictyostelium discoideum* and many other dictyostelid species, solitary amoebae gather and form a multicellular fruiting body in which germ-line spores and somatic stalk cells differentiate, whereas in *Acytostelium subglobosum*, acellular stalks form and all aggregated amoebae become spores. In this study, because most *D. discoideum* genes known to be required for stalk cell differentiation have homologs in *A. subglobosum*, we inferred functional variations in these genes and examined conservation of the stalk cell specification cascade of *D. discoideum* mediated by the polyketide differentiation-inducing factor-1 (DIF-1) in *A. subglobosum*. Through heterologous expression of *A. subglobosum* orthologs of DIF-1 biosynthesis genes in *D. discoideum*, we confirmed that two of the three genes were functional equivalents, while DIF-methyltransferase (*As-dmtA*) involved at the final step of DIF-1 synthesis was not. In fact, DIF-1 activity was undetectable in *A. subglobosum* lysates and amoebae of this species were not responsive to DIF-1, suggesting a lack of DIF-1 production in this species. On the other hand, the molecular function of an *A. subglobosum* ortholog of DIF-1 responsive transcription factor was equivalent with that of *D. discoideum* and inhibition of polyketide synthesis caused developmental arrest in *A. subglobosum*, which could not be rescued by DIF-1 addition. These results suggest that non-DIF-1 polyketide cascades involving downstream transcription factors are required for fruiting body development of *A. subglobosum*.

## INTRODUCTION

Separation of somatic cells from germ-line cells is a crucial event for development of multicellular organisms. How this was achieved during the course of evolution, however, remains an interesting but complex issue (Wolpert and Szathmáry, 2002; Bonner, 2003). Social amoebae are exceptional in that they alternate between unicellular and multicellular phases, serving as excellent model systems for analysis of this issue. They grow as solitary amoebae under sufficient nutrition, but when starved, gather and produce multicellular fruiting bodies consisting of germ-line spores and somatic stalk cells (Raper, 1984). The processes of fruiting body formation and cell differentiation have been most extensively studied in *Dictyostelium discoideum*. In this species, gathered amoebae separate into prestalk cells (anterior 20% of a migratory slug) and prespore cells (posterior 80%), which terminally differentiate as stalk cells and spores, respectively, at the final step of fruiting body formation (Kessin, 2010).

Although most species of social amoebae likewise construct fruiting bodies composed of spores and stalk cells, those in the genus *Acytostelium* form acellular stalks and all aggregated amoebae develop into spores (Hohl et al., 1968; Cavender and Vadell, 2000). Molecular phylogenetic studies indicated that these acellular stalks do not represent an ancestral property, but rather that they resulted from specific loss of cell differentiation ability in this lineage (Swanson et al., 2002; Schaap et al., 2006). To correlate this loss to genomic alterations, the genome of *Acytostelium subglobosum* was analyzed in comparison with other species that form cellular stalks. Unexpectedly, a whole set of *D. discoideum* genes known to be required for stalk cell differentiation were found to have homologs in *A. subglobosum* (H.U., H. Kuwayama and T. Itoh, unpublished data). Furthermore, we recently showed that all gathered amoebae of *A. subglobosum* sequentially express homologous genes for *D. discoideum* prespore genes and prestalk genes, finally undergoing spore encapsulation (Mohri et al., 2013). These results suggest that the molecular machineries for cell-type specification identified in *D. discoideum* are dysfunctional or have been amended in *A. subglobosum*, even though the relevant genes remain. Thus, detailed functional analysis of the gene products is necessary to elucidate the critical differences relevant to the lack of cell differentiation.

**Fig. 1.**
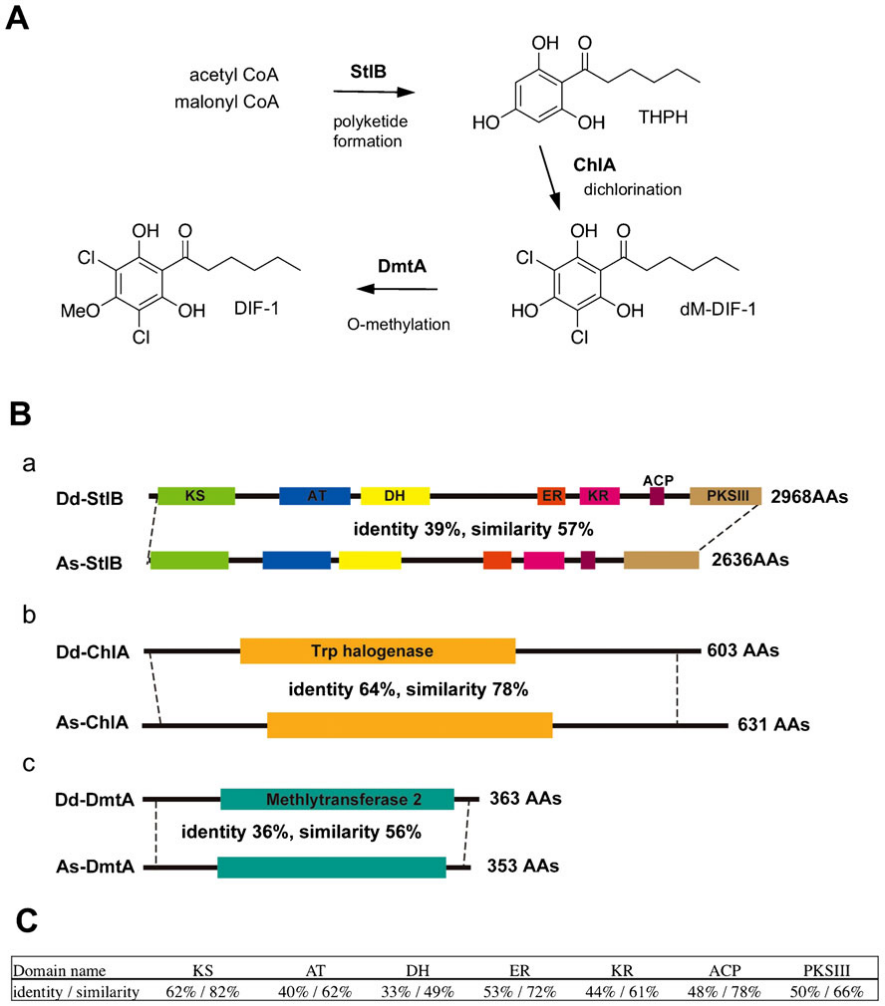
*A. subglobosum* orthologs for DIF-1 synthesis genes. (A) Schematic diagram of DIF-1 biosynthesis. (B) Structures of *Dd-* and *As-stlB* (a), *chlA* (b) and *dmtA* (c) gene products. Domain structures analyzed by the Pfam are shown. The identities and similarities between the amino acid sequences were obtained by BLAST for regions between the dashed lines. (C) Identities and similarities of amino acid sequences between each functional domain of As- and Dd-StlB. KS: beta-ketoacyl synthase, AT: acyltransferase, DH: dehydrase ER: enoyl reductase, KR: ketoreductase, ACP: acyl carrier protein domain, PKSIII: type III PKS domain.

In *D. discoideum*, the polyketide differentiation-inducing factor-1 (DIF-1) was first identified as an inducer of *in vitro* stalk cell differentiation and of expression of a subset of prestalk genes (Morris et al., 1987). It was later shown that the DIF-1 signal is required not for the formation of entire stalk structures but for differentiation of prestalk-cell subtypes, pstO and pstB, which end up in the lower cup and basal disc structures of the fruiting body (Thompson and Kay, 2000b; Saito et al., 2008; Yamada et al., 2011). DIF-1 is produced in prespore cells by a series of enzymatic reactions (Fig. 1A), the first of which is synthesis of (2,4,6-trihydroxyphenyl)-1-hexan-1-one (THPH), catalyzed by the polyketide synthase (PKS) StlB (Austin et al., 2006). THPH is then chlorinated by a flavin-dependent halogenase, ChlA, to yield desmethyl-DIF-1 (dM-DIF-1), and is finally methylated by a specific methyltransferase, DmtA (Neumann et al., 2010; Thompson and Kay, 2000b). In response to DIF-1, transcription factors such as DimA, DimB and MybE translocate from the cytoplasm to the nucleus and bind to upstream regions of the prestalk genes such as *ecmA* and *ecmB* to promote their expression (Thompson et al., 2004; Huang et al., 2006; Zhukovskaya et al., 2006; Fukuzawa et al., 2006).

Although the DIF-1 signal in *D. discoideum* is required for differentiation of lower cups and basal discs but not stalk rods, the function of the DIF-1 cascade in *A. subglobosum* remains of interest because this species lacks entire stalk structures, including the lower cup and basal discs (Mohri et al., 2013). In the present study, we therefore aimed to determine whether the homologous genes for DIF-1 signaling have similar functions in *A. subglobosum* by introducing them into corresponding *D. discoideum* mutants. Our results showed that all *A. subglobosum* homologs except the DmtA gene resumed the mutant defects. It is suspected that DIF-1 signaling itself is inactive in *A. subglobosum* but that a distinct species of polyketide(s) functions in its development.

## MATERIALS AND METHODS

### Isolation of cDNA clones and construction of expression vectors

Total RNA was extracted from the developing sorogens at 16 h after starvation using an RNeasy RNA Extraction Kit (Qiagen) and reverse transcribed with Superscript III Reverse Transcriptase (Life Technologies) to synthesize cDNA. The coding sequences of *Dd-stlB*, *Dd-chlA*, *Dd-dmtA* and *Dd-dimB* were inferred from dictybase (http://dictybase.org) while those of *As-stlB* and *As-chlA* were obtained from the *Acytostelium* Gene Database (AcytoDB, http://acytodb.biol.tsukuba.ac.jp). They were amplified by polymerase chain reaction (PCR) using the primers shown in supplementary material Table S1. The coding sequences corresponding to *As-dmtA* and *As-dimB* were amplified from expressed sequence tag clones (asdv24b18 and asdv14f21, respectively) obtained from NBRP-nenkin (http://nenkin.lab.nig.ac.jp). Amplified fragments were first ligated into the pCR-BluntII-TOPO cloning vector (Life Technologies) for sequence confirmation then subcloned into the expression vector pDM304 (Veltman et al., 2009). In the case of *Dd-* and *As-stlB*, two fragments of coding regions were independently amplified and combined into the expression vector.

### Cell culture and fruiting body formation

*D. discoideum* mutants *stlB-*, *dmtA-*, v31853 (*chlA-*) and *dimB-* were provided by the Dicty Stock Center (http://dictybase.org) and NBRP-nenkin (http://nenkin.lab.nig.ac.jp). These mutants, the wild-type *D. discoideum* strain AX2 and rescued transformants, were cultured in HL5 medium, harvested at the early growth phase and spread onto 1.5% agar plates at appropriate densities. *A. subglobosum* strain LB1 was cultured and harvested as described previously (Mohri et al., 2013) and spread on an agar plate at a density of 9×10^5^ cells/cm^2^ for fruiting body development.

### Cell transformation

Cells were suspended in 400 µl electroporation buffer (10 mM NaPO_4_, 50 mM sucrose, pH 6.1) at 5×10^7^ cells/ml, mixed with 1–20 µg of plasmids and then pulsed with ECM 830 square pulse electroporator (BTX) under the following conditions: 500 V, 100 ms, 15 times with 1-s intervals (Kuwayama et al., 2002). Pulsed cells were then incubated at 21°C for 15 min with 4 µl of 100 mM CaCl_2_ and MgCl_2_, and mixed with 40 ml of HL5. In order to ensure clonal isolation of the primary transformants, the cell suspension was immediately divided into 100 µl aliquots and dispensed into wells of four 96-well culture dishes. The dishes were incubated for 24 h at 21°C followed by the addition of 100 µl of 20 mg/ml G418. The dishes were incubated for a further week until the clones became detectable as cell spots in each well.

### Quantitative RT-PCR

Total RNA was extracted from the appropriate stages of amoebae using an RNeasy RNA Extraction Kit (Qiagen) and first strand cDNA synthesized with Superscript III Reverse Transcriptase (Life Technologies). For quantification, real-time RT-PCR was performed using an ABI 7900HT Sequence Detection System (Applied Biosystems). Amplifications were carried out using Power SYBR Green PCR Master Mix (Applied Biosystems). The concentration of the templates was adjusted by amplification using primers for *Dd-Ig7* (mitochondrial large ribosomal RNA) or *As-elp3* (a subunit of the RNA polymerase II elongator complex). The primer sequences are shown in supplementary material Table S2.

### Extraction of DIF-1 fraction from amoebae

Amoebae of *D. discoideum* (dry weight 1.13 g) and *A. subglobosum* (dry weight 2.02 g) at the 16 h stage of fruiting body formation were extracted with 100 ml methanol at room temperature to give methanol extracts. The extracts were then partitioned with 100 ml of ethyl acetate and 100 ml water, and the ethyl acetate layer was evaporated using a rotary evaporator to give an ethyl acetate soluble. This soluble was chromatographed over 1.5 g silica gel and the column eluted with hexane–ethyl acetate solutions at increasing polarity to give five fractions.

### DIF bioassay

An *in vitro* stalk cell induction assay was performed as described previously (Thompson and Kay, 2000a). Washed cells were suspended in stalk medium (2-(N-morpholino) ethane sulfonic acid, 10 mM KCl, 2 mM NaCl, 1 mM CaCl_2_, pH 6.2) with 5 mM cAMP at 2.5×10^4^ cells/ml and incubated at 22°C for 24 h. After washing twice with KK2 (20 mM K_2_HPO_4_/KH_2_PO_4_, pH 6.8), cells were incubated with stalk medium containing various concentrations of DIF-1 or fractions of HPLC. As reported, addition of DIF-1 to the monolayer culture of *D. discoideum* amoebae previously stimulated with cAMP induced differentiation of amoebae into vacuolated stalk cells (Morris et al., 1987). Cells were observed by phase-contrast microscopy to determine the number of vacuolated differentiated stalk cells and RNA extracted for quantification of gene expression.

## RESULTS

### Identification of orthologs to *D. discoideum* genes for DIF-1 synthesis in *A. subglobosum*

To identify *A. subglobosum* orthologs of *stlB*, *chlA* and *dmtA*, we performed a TBLASTN search against the *A. subglobosum* genome in AcytoDB using the amino acid sequences of *D. discoideum* gene products and reconstituted the coding sequences from the top hits. The exon–intron boundaries were verified using the expressed sequence tag sequence for *dmtA* and by RT-PCR for *stlB* and *chlA* homologs. Since a reverse search against the *D. discoideum* protein database indicated that they were indeed orthologs of *stlB*, *chlA* and *dmtA*, we named them *As-stlB*, *As-chlA* and *As-dmtA* (DDBJ ID: AB902921, AB902922, AB902923), respectively. To avoid confusion, *stlB*, *chlA* and *dmtA* are designated *Dd-stlB*, *Dd-chlA* and *Dd-dmtA*, respectively, where appropriate. Functional domains found in the *D. discoideum* gene products are all conserved in their *A. subglobosum* counterparts (Fig. 1B,C). As-StlB contained a complete set of the multiple functional domains required for polyketide synthase activity of Dd-StlB in the exact order.

### Temporal expression patterns of *A. subglobosum* orthologs for DIF-1 synthesis genes

**Fig. 2.**
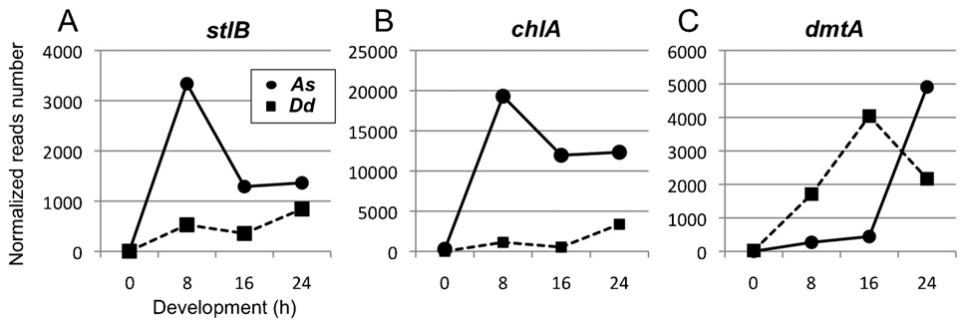
Temporal expression patterns of *A. subglobosum* (circles and solid lines) and *D. discoideum* (squares and dashed lines) *stlB* (A), *chlA* (B) and *dmtA* (C). Expression data of each gene were obtained from large-scale RNA sequencing analysis provided by dictyexpress (http://dictyexpress.biolab.si) and our ongoing RNA sequencing project of *A. subglobosum*. Total reads corresponding with each gene were normalized with gene lengths and total read numbers from whole RNA at each developmental time point.

Temporal expression data corresponding to *As-stlB*, *As-chlA* and *As-dmtA* were extracted from massive mRNA sequencing. Although morphologically different, the time course of *A. subglobosum* fruiting body formation is similar to that of standard *D. discoideum* strains; mound formation at around 8 h and culmination from 16 h to 24 h after starvation (Mohri et al., 2013). Thus, we examined *A. subglobosum* gene expression at these stages in comparison with that of *D. discoideum*. As shown in Fig. 2, the expression of all three genes for DIF-1 production was negligible at the growth phase and increased during development. Expressions of *As-stlB* and *As-chlA* were very similar; that is, both increased soon after starvation, peaked at 8 h, and maintained a considerably high level. On the other hand, expression of *As-dmtA* was negligible until 16 h and increased dramatically at 24 h.

When the expression data of the *Dd* counterparts (downloaded from Parikh et al., 2010) were plotted in the same graphs after appropriate normalization, remarkable differences were observed between the orthologous pairs. Expression levels of *As-stlB* and *As-chlA* were found to be much higher than their *D. discoideum* counterparts. The latter peaked at 24 h, although levels of *A. subglobosum* mRNAs were still higher at this time. Peak levels of expression were comparable between *As-dmtA* and *Dd-dmtA*, but their expression patterns were dissimilar. The rapid increase in *Dd-dmtA* mRNA during early development was completely missing in *As-dmtA*. These dissimilarities in expression, both in levels and temporal patterns, suggest that the *A. subglobosum* homologs do not play analogous roles with their *D. discoideum* counterparts during fruiting body formation.

### Functional analysis of *A. subglobosum* orthologs for DIF-1 synthesis enzymes

**Fig. 3.**
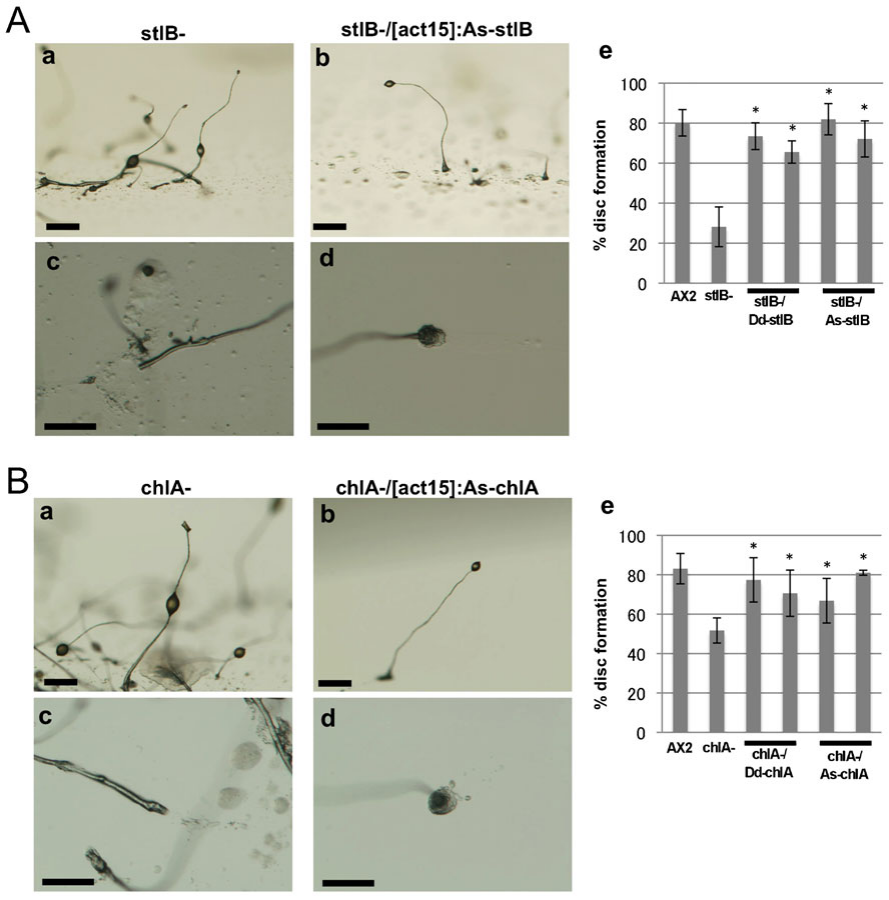
Phenotypic rescue of *D. discoideum stlB* (A) and *chlA* (B) null mutants with overexpression of their *A. subglobosum* orthologs. Mutants and transformants were developed on agar and morphologies of fruiting bodies observed at 36 h. Representative morphologies of whole fruiting bodies (a,b) and roots of sorocarps (c,d) from mutants (a,c) and those transformed with the corresponding *A. subglobosum* gene (b,d). Percentages of fruiting bodies in the null mutant and transformants with basal discs are summarized in panel e. Error bars indicate the mean ± standard deviation. *p<0.05 vs null mutant (t-test). Scale bars: 200 µm.

**Fig. 4.**
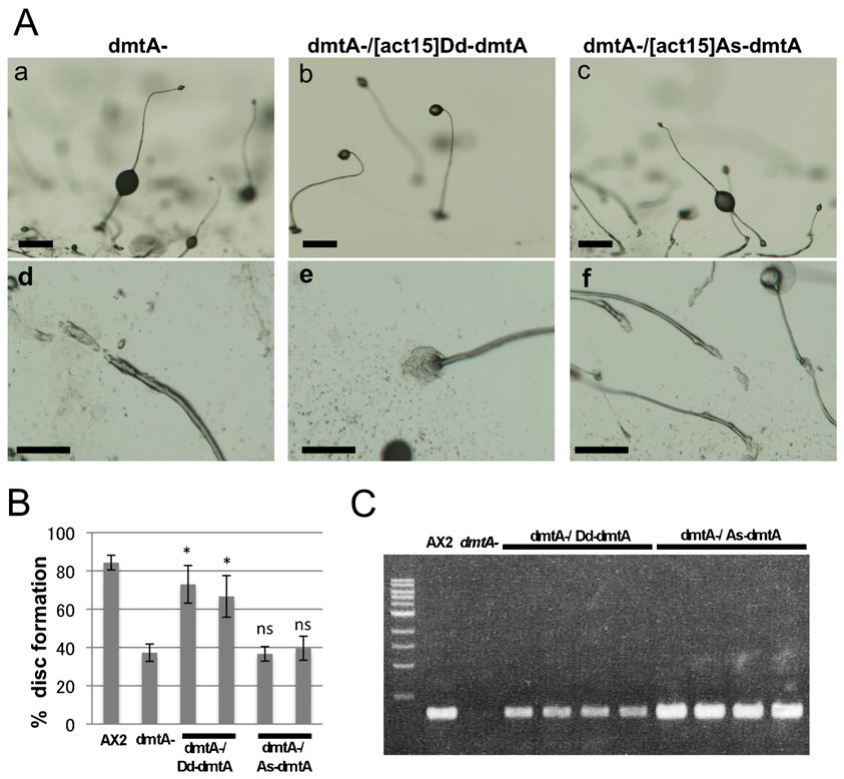
Rescue experiments of *D. discoideum dmtA* null mutants with overexpression of their *A. subglobosum* orthologs. *dmtA-* and transformants were developed on agar and morphologies of fruiting bodies observed at 36 h. (A) Representative morphologies of whole fruiting bodies (a–c) and roots of sorocarps (d–f) from *dmtA*- mutants (a,d) and those transformed with *Dd-* (b,e) or *As-dmtA* (c,f). (B) Summary of the percentages of fruiting bodies with basal discs. Error bars indicate the mean ± standard deviation. *p<0.05, ^ns^non-significant vs *dmtA-* (t-test). (C) Expression of transformed genes was verified by RT-PCR. cDNA of *Dd-* (six lanes from the left) and *As-dmtA* (four lanes from the right) were amplified. Scale bars: 200 µm.

To determine whether the functions of the *A. subglobosum* orthologs were conserved, they were expressed in corresponding *D. discoideum* null mutants and the resulting phenotypes examined. As previously reported, the mutants with defects in DIF-1 production showed “DIF-less phenotypes” such as fragmented slug body, prostrate sorocarps and slipped down sorus attributed to the reduction in pstO and pstB cells in the slug and in the basal disc and lower cup structures of the fruiting body where these cells should have populated (Thompson and Kay, 2000b; Saito et al., 2008; Yamada et al., 2011). These phenotypes of the *stlB* and *chlA* null mutants were fully restored by expression of *As-stlB* and *As-chlA*, respectively, under the constitutive act15 promoter as by *D. discoideum* counterparts (Fig. 3; supplementary material Figs S1, S2). However, while *Dd-dmtA* expression restored the DIF-less phenotypes in the *dmtA* mutant, expression of *As-dmtA* did not, (Fig. 4A,B; supplementary material Fig. S3A). The possibility of unsuccessful *As-dmtA* expression in the transformants was excluded by RT-PCR amplification of mRNA prepared from the *As-dmtA* introduced mutants (Fig. 4C). Thus, these findings show that the function of the first two enzymes in the DIF-1 biosynthesis pathway, StlB and ChlA, are conserved across species, while that of the *A. subglobosum* homolog of the last enzyme, As-DmtA, is doubtful. Since all three residues required for catalytic activity of O-methyltransferase are conserved but those for S-adenosylmethionine binding are not completely conserved in As-dmtA (6 out of 8) (Zubieta et al., 2001), this insufficient complementation may be due to incomplete conservation of the latter or unidentified residues required for substrate recognition (supplementary material Fig. S3B).

### Examination of DIF-1 signaling in *A. subglobosum*

**Fig. 5.**
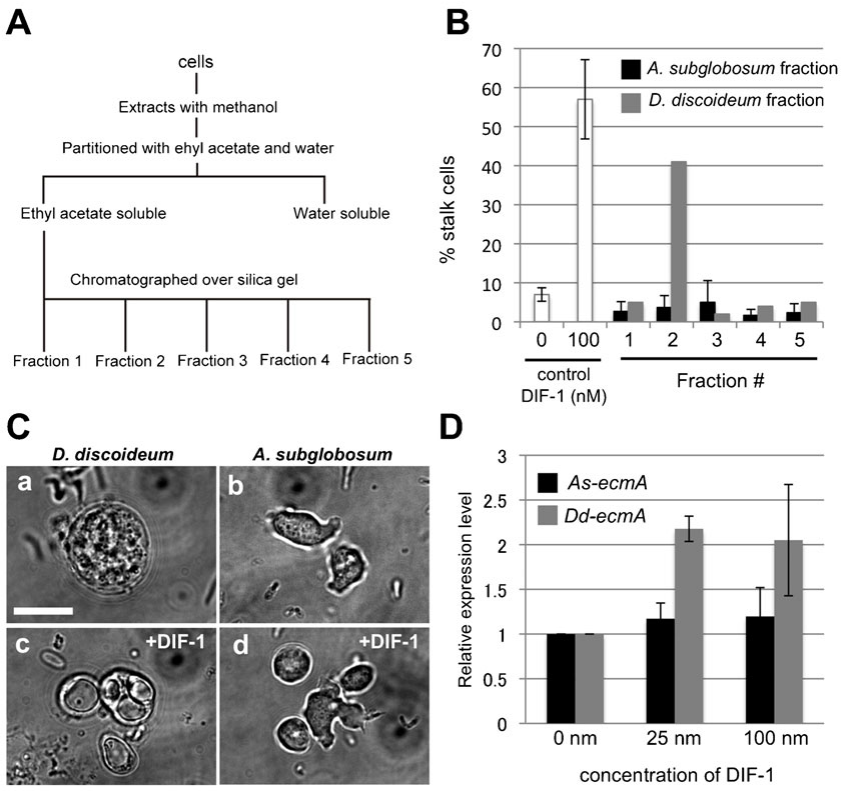
Extraction of DIF and reaction of *A. subglobosum* against DIF-1. (A) Flowchart showing the experimental procedure for separating DIF-1 containing fractions. *A. subglobosum* 2.02 g or *D. discoideum* 1.13 g were used. (B) DIF bioassay with fractions of cell extracts. DIF-1 or fractions obtained from *A. subglobosum* (black bar) or *D. discoideum* (gray bar) cells were added to the *D. discoideum* amoebae after stimulation with 5 mM cAMP. Percentages of vacuolated stalk cells were examined 24 hours after addition. (C) Morphological changes of amoebae in the presence of DIF-1. *D. discoideum* (a,c) and *A. subglobosum* (b,d) amoebae stimulated with cAMP were cultured for 24 h in the absence (a,b) or presence (c,d) of 100 nM of DIF-1. (D) Total RNA was extracted from cells cultured with 0–100 nM DIF-1 and expression levels of *As-* (black bar) and *Dd-ecmA* (gray bar) were examined with real-time RT-PCR. Levels of mRNA are shown relative to the 0 nM samples. Error bars indicate the mean ± standard deviation. Scale bar: 10 µm.

Since we were unable to confirm whether the entire DIF-1 biosynthesis pathway is active in *A. subglobosum*, we attempted to detect DIF-1 activity in developing *A. subglobosum*. To this end, a methanol extract from *A. subglobosum* cells was partitioned with ethyl acetate and fractionated by HPLC (Fig. 5A) then used for the DIF bioassay described in Materials and Methods. Addition of DIF-1 to a monolayer culture of *D. discoideum* amoebae that were pre-stimulated with cAMP induced differentiation of amoebae into vacuolated stalk cells as shown in Fig. 5C. HPLC fraction number 2 from the *D. discoideum* sample showed a significant level of stalk cell-inducing activity; however, neither the corresponding fraction nor neighboring fractions from the *A. subglobosum* sample showed any inducing activities (Fig. 5B).

Next, we performed a DIF bioassay against *A. subglobosum* amoebae to determine whether *A. subglobosum* cells show any response to DIF-1. In contrast to *D. discoideum* cells, no morphological changes were observed in *A. subglobosum* (Fig. 5C). To detect molecular changes, we determined the expression level of *ecmA* in DIF-1 stimulated amoebae of both species by quantitative RT-PCR. While the expression of *Dd-ecmA* showed a more than 2-fold increase in the presence of 25 nM DIF-1, that of *As-ecmA* was unaffected even at 100 nM DIF-1 (Fig. 5D). From these results, we concluded that *A. subglobosum* does not employ DIF-1 signaling during development.

### Functional conservation of a DIF-1 responsive gene in *A. subglobosum*

**Fig. 6.**
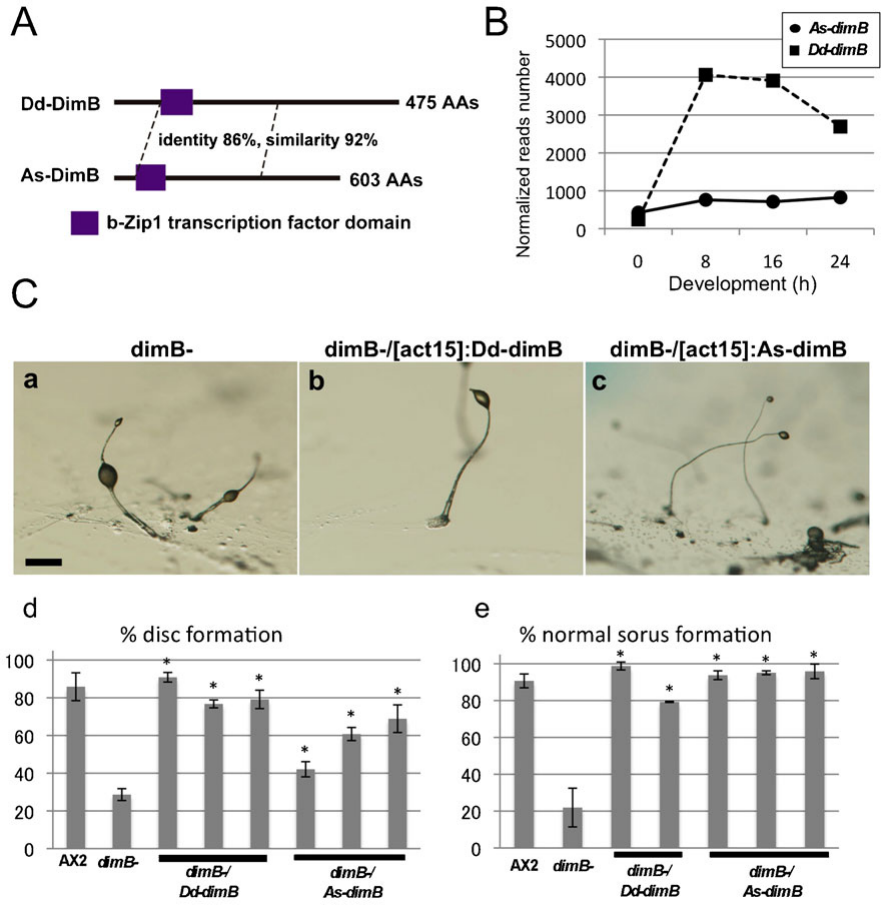
Phenotypic rescue of *D. discoideum dimB* null mutants with overexpression of *A. subglobosum dimB* orthologs. (A) Structures of *Dd-* and *As-dimB* gene products. Domain structures analyzed by Pfam, and identities and similarities between the amino acid sequences obtained by BLAST are shown. (B) Temporal expression patterns of *As-* (circles and solid line) and *Dd-* (squares and dashed line) *dimB*. Expression data of each gene were obtained from large-scale RNA sequencing analysis. (C) Morphologies of fruiting bodies of *dimB* null mutants (a) and those transformed with *Dd-* (b) and *As-dimB* (c). Percentages of fruiting bodies with basal discs (d) and normal sori on the top of sorocarps (e) are summarized. Error bars indicate the mean ± standard deviation. *p<0.05 vs *dimB-* (t-test). Scale bar: 200 µm.

Since the lack of both DIF-1 production and responsiveness in *A. subglobosum* raised questions about the downstream cascade found in *D. discoideum*, we identified an *A. subglobosum* ortholog of *dimB* (DDBJ ID: AB902924), a DIF-1 responsive *D. discoideum* transcription factor that has been extensively analyzed, and examined its functional conservation in the same way as for DIF-1 synthesis genes. As-DimB was shown to possess a functional domain for DNA binding and dimerization highly similar to that of Dd-dimB (Fig. 6A). However, the expression patterns of *Dd-dimB* and *As-dimB* were very dissimilar (Fig. 6B). While expression of *Dd-dimB* increased soon after starvation and maintained high levels until 16 h, that of *As-dimB* did not increase significantly throughout development (Fig. 6B). The DIF-less phenotypes of the *dimB* null mutant were clearly recovered by constitutive expression of *As-dimB*, indicating that the function of As-DimB is equivalent to that of Dd-DimB (Fig. 6C).

### DIF-1 could not rescue phenotypes of PKS depression in *A. subglobosum*

**Fig. 7.**
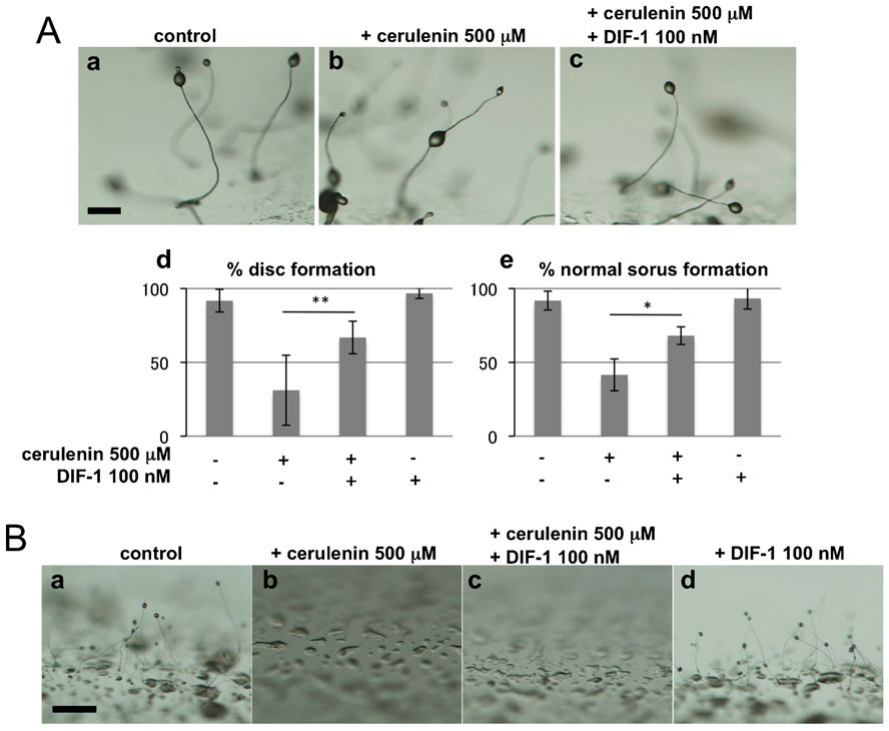
Effects of polyketide synthase inhibition on fruiting body formation of *D. discoideum* (A) and *A. subglobosum* (B). (A) *D. discoideum* cells were cultured on agar with or without 500 µM cerulenin and/or 100 nM DIF-1 and morphologies of fruiting bodies were observed at 36 h (a–c). Percentages of fruiting bodies with basal discs (d) and normal sori on the top of sorocarps (e) are summarized. Error bars indicate the mean ± standard deviation. *p<0.05, **p<0.1 (t-test). (B) *A. subglobosum* cells were developed on agar with or without 500 µM cerulenin and/or 100 nM DIF-1. Scale bars: 200 µm.

Even though DIF-1 is unlikely to control *A. subglobosum* development, it is highly possible that its precursors are synthesized and the downstream cascades active, suggesting bypass polyketide signaling operates in this species. To examine this possibility, we analyzed the effects of the PKS inhibitor cerulenin, which inhibits DIF-1 synthesis in *D. discoideum* (Kay, 1998). When *D. discoideum* cells were developed on agar plates containing 500 µM cerulenin, the resulting sorocarps showed DIF-less phenotypes, while addition of 100 nM DIF-1 rescued the defects, although not completely (Fig. 7A). Depression of other polyketides or fatty acid synthesis is one possible reason for this incomplete rescue. On the other hand, when *A. subglobosum* cells were incubated on agar containing cerulenin, development was halted at the aggregation stage and no sorogens emerged. Interestingly, this inhibition was not rescued by the addition of 100 nM DIF-1 (Fig. 5B). These results indicate that DIF-1 is not required for post-aggregation development of *A. subglobosum* fruiting body formation and suggest that other types of polyketide are required.

## DISCUSSION

The data presented in this study strongly suggest that DIF-1 signaling is not involved in fruiting body formation of the stalk cell-less species *A. subglobosum*. However, the narrow possibility of an alternative explanation remains. That is, unsuccessful complementation of *Dd-dmtA* disruption by *As-dmtA* might have arisen from species-specific differences in protein structure. Moreover, the failure of DIF-1 detection and unresponsiveness to exogenous DIF-1 may simply reflect methodological inappropriateness. Even so, details of latent DIF-1 signaling in *A. subglobosum* should remain quite different from that in *D. discoideum*. We subsequently showed that DIF-1 involving molecular machinery for fruiting body formation varies largely among social amoeba with and without cell differentiation. This study illustrates that in addition to analysis of the repertoires of homologues between genomes, comparison of molecular functions among homologous gene products is also required to understand the causes of morphological variations between species.

### Relevance of the lack of a DIF-1 cascade and the loss of stalk cell subtypes

Although the presence of a lower cup structure supporting the sorus has not been documented except for *D. discoideum*, the supportive structures at the sorocarp roots represented by the basal disc have been observed in various species. Recent phylogenetic studies indicate that these structures are mostly associated with an evolutionarily advanced group (group 4) with only a few exceptions, coinciding with the larger size of sori (Schaap et al., 2006). A DIF-1 cascade is required for differentiation of these supportive structures in *D. discoideum*; thus, the lack of DIF-1 production in *A. subglobosum* observed in this study raises the possibility that the evolutionarily advanced species developed the DIF-1 signaling system to generate these supportive structures. This possibility is supported by a report that *D. purpureum* in group 4, which has a special supportive structure at the stalk root, produces a DIF-1-like molecule, and by the fact that *D. purpureum dmtA* is able to rescue defects of *D. discoideum dmtA* mutants, in contrast to the case of *A. subglobosum* shown in this study (Motohashi et al., 2012).

### Supposed non-DIF-1 polyketide cascades in *A. subglobosum*

Since the temporal expression patterns of *As-stlB* and *As-chlA* were mutually similar with those of *D. discoideum* and since they were shown to be functionally equivalent to their *D. discoideum* counterparts, it is likely that they work together in a synthetic pathway to generate dM-DIF-1. However, whether dM-DIF-1 is the final product or subsequently modified by other enzymes remains unknown. Accordingly, it is noteworthy that accumulation of dM-DIF-1 causes loss of pstO cells in *Dd-dmtA* deletion mutants, suggesting physiological roles of DIF-1 precursor(s) (Thompson and Kay, 2000b; Saito et al., 2008). Whether the dM-DIF-1 is actually synthesized in *A. subglobosum* cells and how it functions during fruiting body formation are interesting questions for future studies. Our experiments using cerulenin demonstrate the requirement of non-DIF-1 polyketides for post aggregation development of *A. subglobosum* fruiting bodies. dM-DIF-1 or subsequently modified polyketides would work in this process. However, since cerulenin also inhibits fatty acid synthase, it is also possible that certain fatty acids are required for fruiting body formation of *A. subglobosum*.

The *A. subglobosum* ortholog of DIF-1 responsive transcription factor *dimB* was able to rescue defects of *dimB*- mutants of *D. discoideum*, indicating that As-DimB works under DIF-1 signals in *D. discoideum* and that its activity in transcription of stalk-specific genes is conserved. Since *As-ecmA*, which is highly homologous to both *Dd-ecmA* and *ecmB*, was expressed in spore-committing cells in elongating sorogens (Mohri et al., 2013), we speculate that As-DimB may function as a transcription factor for *As-ecmA* and other genes required for acellular stalk formation. However, it should be mentioned that the introduced upstream regulatory region of *As-ecmA* combined with *lacZ* did not work in *D. discoideum* stalk cells (data not shown). The functional equivalent of DimB protein may not be sufficient for *As-ecmA* expression. The molecular machinery required for the DIF-1 response in *D. discoideum* has yet to be fully elucidated; for example, how cells receive the DIF-1 molecule remains unclear. This unknown component of the cascade is perhaps changed in *A. subglobosum* and transcription factors involving As-DimB may be responsive to the non-DIF-1 polyketide(s) described above.

## Supplementary Material

Supplementary Material
