# Supplementary material for: Defects in the synthetic pathway prevent DIF-1 mediated stalk lineage specification cascade in the non-differentiating social amoeba, Acytostelium subglobosum
Source: Biol Open. 2014 May 29;3(6):553–60. doi: 10.1242/bio.20148359 (PMC4058090; doi:10.1242/bio.20148359)
Supplement: Supplementary Material [file supp_3_6_553__index.html]

Defects in the synthetic pathway prevent DIF-1 mediated stalk lineage specification cascade in the non-differentiating social amoeba, Acytostelium subglobosum — Defects in the synthetic pathway prevent DIF-1 mediated stalk lineage specification cascade in the non-differentiating social amoeba, Acytostelium subglobosum — Supplementary Material 

# Defects in the synthetic pathway prevent DIF-1 mediated stalk lineage specification cascade in the non-differentiating social amoeba, *Acytostelium subglobosum*

## bio.20148359 Supplementary Material

**Files in this Data Supplement:**

- Supplementary Material - Kurato Mohri et al. doi: 10.1242/bio.20148359
