# Supplementary material for: Defects in the synthetic pathway prevent DIF-1 mediated stalk lineage specification cascade in the non-differentiating social amoeba, Acytostelium subglobosum
Source: Biol Open. 2014 May 29;3(6):553–60. doi: 10.1242/bio.20148359 (PMC4058090; doi:10.1242/bio.20148359)
Supplement: Supplementary Material [file supp_bio.20148359_bio.20148359-s1.pdf]

 Biology Open (2014) 000, 1–8 doi:10.1242/bio.20148359

Supplementary Material

Kurato Mohri et al. doi: 10.1242/bio.20148359

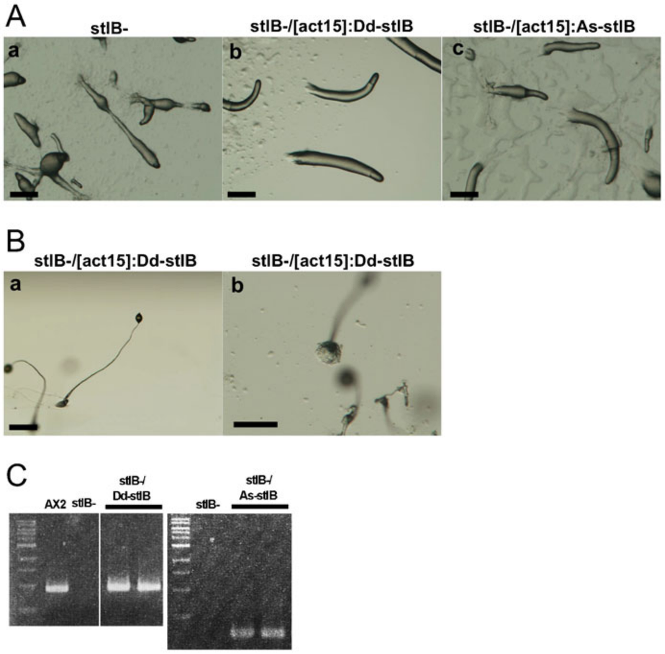

Fig. S1. Phenotypic rescue of *D. discoideum stlB* null mutants with overexpression of *Dd-* or *As-stlB*. (A) Mutant slugs at the 16-h stage of development (a) and those transformed with *Dd-* (b) or *As-stlB* (c). (B) Representative morphologies of whole fruiting bodies (a) and roots of sorocarps (b) of *stlB-* mutants transformed with *Dd-stlB*. (C) Expression of transformed genes was verified by RT-PCR. cDNA of *Dd-* and *As-stlB* was amplified. Scale bars: 200 μm.

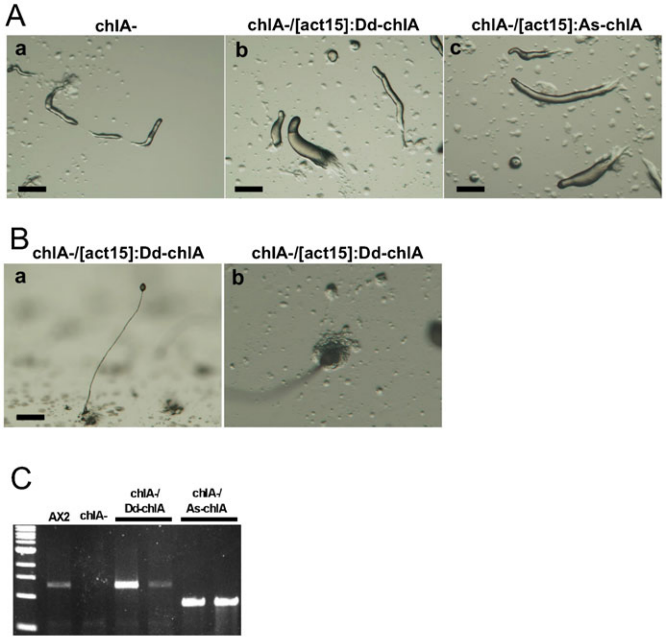

Fig. S2. Phenotypic rescue of *D. discoideum chlA null* mutants with overexpression of *Dd-* or *As-chlA*. (A) Mutant slugs at the 16-h stage of development (a) and those transformed with *Dd-* (b) or *As-chlA* (c). (B) Representative morphologies of whole fruiting bodies (a) and roots of sorocarps (b) of *chlA-* mutants transformed with *Dd-chlA*. (C) Expression of transformed genes was verified by RT-PCR. cDNA of *Dd-* and *As-chlA* was amplified. Scale bars: 200 μm.

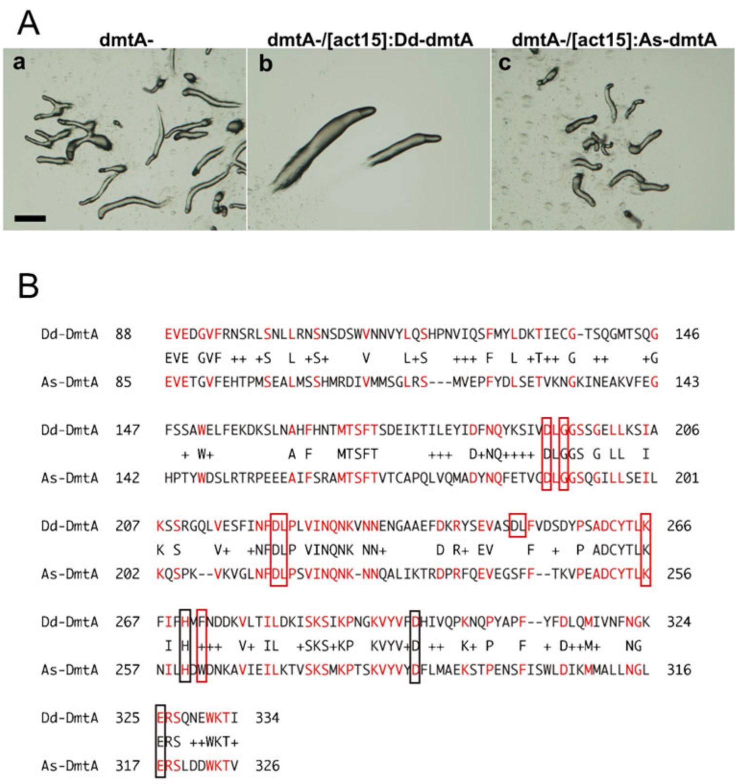

Fig. S3. Phenotypic rescue of *D. discoideum dmtA null* mutants with overexpression of their *A. subglobosum* orthologs. (A) *dmtA-* slugs at the 16-h stage of development (a) and those transformed with *Dd-* (b) or *As-dmtA* (c). (B) Alignment of amino acid sequences of methyltransferase domains of Dd- and As-DmtA. Encircled residues are those thought to be required for S-adenosylmethionine binding (red) and for catalytic activity (black). Scale bar: 200 μm.

Biology Open (2014) 000, 1–8 doi:10.1242/bio.20148359

Table S1. PCR primers used for cDNA cloning

Primer	Sequence (5′ to 3′)
As-stlB5′ forward BamHI	GTTCGGATCCATGAGCAGAAATCAACAACCTATCG
As-stlB5′ reverse SpeI	AACTACTAGTACGGTCTCGCGAAGGTTCGACCACCAG
As-stlB3′ forward	ACTCCACCACTCTGGGCAAGCTGATCGATGC
As-stlB3′ reverse SpeI	TACGACTAGTTTATTCGGCATTGATCATTCTGAGC
As-chlA forward BamHI	GTTCGGATCCATGGACCCAACGGATATCCAATCAG
As-chlA reverese SpeI	TACGACTAGTTTACATTGATGACTCCCAAGGCACG
As-dmtA forward BglII	GTTCAGATCTATGGAAGCTAAAGCAACTGAATCTG
As-dmtA reverse SpeI	TACGACTAGTTTACTTGCCAAAGATGACCAGAGTTG
As-dimB forward BglII	TTAGAGATCTAAAAAAATGACCAAGAAGAATCAACAGCAAC
As-dimB reverse SpeI	TTACACTAGTGTAGTATCCAGTGTTGCTCTGGATTGG
Dd-stlB5′ forward BamHI	GTTCGGATCCATGAACAACAACAAAAGTATAAACG
Dd-stlB5′ reverse SpeI	AAGTACTAGTTGATAGCAGATTGTAAAACTGCATC
Dd-stlB3′ forward	ATTACCAGGTAGAAGATGGAAACTTG
Dd-stlB3′ reverse XbaI	TTGGTCTAGATTATTTAATTAATTTAAATAAAATTGCTTCAATTG
Dd-chlA forward BglII	GTTCAGATCTATGGATACAAATATTATTAATCATTATG
Dd-chlA reverse SpeI	TACGACTAGTTTAATACTTCCAATCAACAATATTA
Dd-dmtA forward BamHI	GTGCGGATCCATGATTTCAAATTCGGATTGTGATG
Dd-dmtA reverse XbaI	TACCTCTAGACTATTGTTTTAAAGATAATTCAATG
Dd-dimB forward BamHI	TTCAGGATCCAAAAAAATGAATCAATTTTATCAATCTACCACTGG
Dd-dimB reverse SpeI	TTACACTAGTTTATTGTCTCGAAGGTTGTTGTTGGTGG

Table S2. PCR primers used for expression analysis

Primer	Sequence (5′ to 3′)
RT-PCR	
As-stlB forward	ATGGAAGCTGTTGGCAACAAGC
As-stlB reverse	TGGAACTGGAGCGAGAACTTGG
As-chlA forward	CAACAAGCATTATGACGTCGTG
As-chlA reverse	GTTCTCGATCAGGTAGTCCTGG
As-dmtA forward	GTGATGGATTTGGCTGATGGAC
As-dmtA reverse	ATTCTTGACGGTCTCGGACAGG
As-dimB forward	CAACAGATGCAACAGCAACAAC
As-dimB reverse	CTTGGCTTCCATCATCATTGTG
Dd-stlB forward	ATCATCACCAAATCCAATCGTTGTAC
Dd-stlB reverse	AAATTACTGTTGCCATTGACGAATCG
Dd-chlA forward	AGTGAAGTTGAATCTGGTGCCG
Dd-chlA reverse	CATAATAATCATTATTTGGAGTACCAATC
Dd-dmtA forward	AAGTGGAGGATGGAGTCTTCAG
Dd-dmtA reverse	GAACCACCCAAATCAACGATTG
Dd-dimB forward	AATGGAGACGGATCAGATGATG
Dd-dimB reverse	TTTCTCGACTTCTCTCTCGAC
Quantitative RT-PCR	
As-ecmA forward	CCTTTGACAGCTGTAGCAAC
As-ecmA reverse	ATTGTCGGGACAGCAAGTTC
Dd-ecmA forward	CTCAACACCAGTTTCATGTCC
Dd-ecmA reverse	ATTCATTGGGACTTCGATGC
Dd-Ig7 forward	GGCGGTAACCTTCTAAAGAG
Dd-Ig7 reverse	TTGCGTCTCTGATCGACTTG
As-elp3 forward	GTATCCAGGAGGTGCATCAC
As-elp3 reverse	GGTCCTCATACGACAGGAAG

Biology Open
